# Purine nucleoside antibiotics: recent synthetic advances harnessing chemistry and biology

**DOI:** 10.1039/d3np00051f

**Published:** 2024-01-10

**Authors:** Jonas Motter, Caecilie M. M. Benckendorff, Sarah Westarp, Peter Sunde-Brown, Peter Neubauer, Anke Kurreck, Gavin J. Miller

**Affiliations:** a Chair of Bioprocess Engineering, Institute of Biotechnology, Faculty III Process Sciences, Technische Universität Berlin Ackerstraße 76 D-13355 Berlin Germany; b School of Chemical and Physical Sciences and Centre for Glycoscience, Keele University Keele Staffordshire ST5 5BG UK g.j.miller@keele.ac.uk; c BioNukleo GmbH Ackerstraße 76 13355 Berlin Germany anke.kurreck@bionukleo.com

## Abstract

Covering: 2019 to 2023

Nucleoside analogues represent one of the most important classes of small molecule pharmaceuticals and their therapeutic development is successfully established within oncology and for the treatment of viral infections. However, there are currently no nucleoside analogues in clinical use for the management of bacterial infections. Despite this, a significant number of clinically recognised nucleoside analogues are known to possess some antibiotic activity, thereby establishing a potential source for new therapeutic discovery in this area. Furthermore, given the rise in antibiotic resistance, the discovery of new clinical candidates remains an urgent global priority and natural product-derived nucleoside analogues may also present a rich source of discovery space for new modalities. This Highlight, covering work published from 2019 to 2023, presents a current perspective surrounding the synthesis of natural purine nucleoside antibiotics. By amalgamating recent efforts from synthetic chemistry with advances in biosynthetic understanding and the use of recombinant enzymes, prospects towards different structural classes of purines are detailed.

## Abstract 2

This Highlight offers a perspective on recent strategies for synthesising purine nucleoside antibiotics, an untapped source of natural products for developing novel antimicrobial agents.

## Graphical abstract

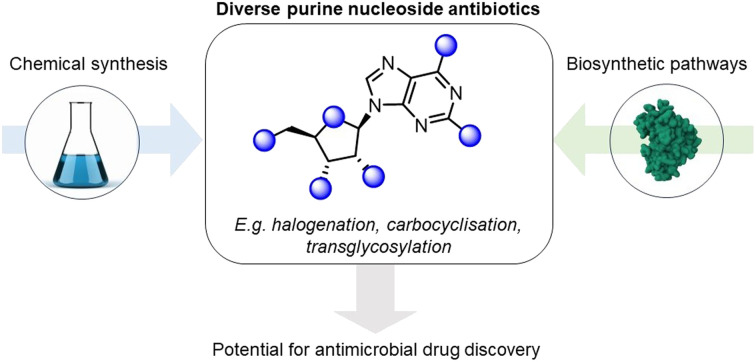

## 1. Introduction

Nucleoside antibiotics are a diverse subset of microbial natural products, whose evolution has led to a variety of unusual structural characteristics which mimic or present motifs related to the component nucleosides and nucleotides of nucleic acids.^1–3^ Traditionally, structural analogues of nucleosides and nucleotides have been investigated for anticancer and/or antiviral therapeutic potential.^4–10^ However, an often-overlooked facet of these molecules and related microbial natural products is their antibacterial activity. There has been a recent increase in interest in nucleoside analogues as a source for novel antibacterial exploration,^11,12^ compounded by the rise of antibiotic resistance within standard intervention therapies.^13^

**Fig. 1:**
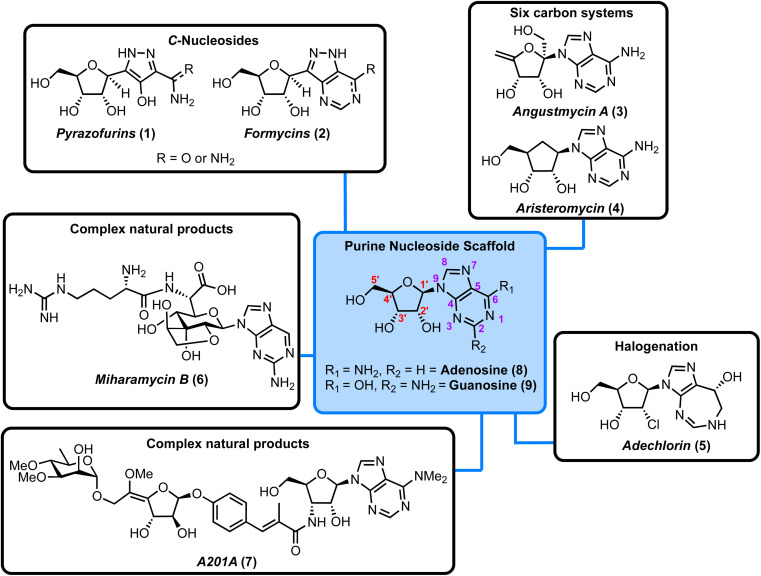
Overview of the purine nucleoside natural products considered in this Highlight, along with the chemical structure of the canonical purine nucleosides, adenosine, and guanosine (blue box, red numbering for ribose ring and purple for heterocycle).

Depending on their constituent heterocyclic base, nucleoside antibiotics can be divided into pyrimidine and purine classes and the structures of the canonical adenine- and guanine-containing systems are highlighted in Fig. 1 (blue box). These nucleosides consist of a sugar core (d-ribose) linked to the heterocyclic nucleobase *via* a β-*N*-glycosidic bond. When combined with either adenine (6-aminopurine) or guanine (2-amino-6-hydroxypurine) the structures comprise adenosine (8) and guanosine (9), respectively. Modifications to these scaffolds present the basis for structurally diverse nucleoside antibiotics, with examples incorporating *C*-glycosides (β-*C*-glycosidic linkage), ribose ring halogenation and the attachment of peptides, illustrated further in Fig. 1.

Traditionally, chemical synthesis has served as the primary tool to access biologically relevant, structurally defined, and homogenous purine nucleoside antibiotics. However, due to their structural complexity, such syntheses are often challenging and can require multi-step routes to successfully prepare a given target.^14,15^ This can lead to low overall yields of material. To overcome these issues, considerable progress has been made in developing innovative synthetic solutions (*vide infra*), but also towards elucidating and understanding the biosynthetic pathways that produce nucleoside antibiotics. Availability of recombinant enzymes that operate within such pathways can provide an alternative, biocatalytic approach towards the synthesis of relevant targets. This has been reviewed recently for pyrimidine nucleoside antibiotics.^16^

In this Highlight we present and synergise advances from 2019 onwards surrounding the understanding of biosynthetic pathways/enzymes and related synthetic chemistry platforms that can deliver purine nucleoside antibiotics. Readers interested in a comprehensive overview of over a hundred isolated purine nucleoside antibiotics and their bioactivity are encouraged to visit Inoso's reports from 1988 and 1991.^1,2^Table 1 presents the antimicrobial activity of the nucleosides discussed herein.

**Table 1:** The antibiotic activity of nucleosides discussed in this Highlight^a^

Antibiotic		Antimicrobial activity	Reference
*C*-Nucleosides	Pyrazofurins	Broad-spectrum antiviral, high toxicity (mice LD_50_: 10 mg kg^−1^) by blocking *de novo* pyrimidine synthesis; human trials for cancer treatment	17 and 18
	Formycins	Inhibition of *X. oryzae* at 6 mg mL^−1^ and *Mycobacterium* 607 at 6 mg mL^−1^; high toxicity (mice LD_50_: 250–500 mg kg^−1^, cumulative toxicity)	19
Six-carbon framework	Angustmycin A and C	Inhibitor of GMP synthesis in Gram-positive bacteria, *in vitro* inhibition of *S. aureus* at >10 mg mL^−1^; *in vivo* activity in infected mice (CD_50_ angustmycin C: 12.8 mg kg^−1^)	20 and 21
	Aristeromycin	SAH hydrolase inhibitors, effective against *X. oryzae* and *P. oryzae in vitro* (MIC: 5 mg mL^−1^) and in rice plants *in vivo*	22 and 23
Halogenated	Adechlorin	Adenosine deaminase inhibitor, highly effective against *E. faecalis* with MIC: 0.005 μg mL^−1^	24
	Nucleocidin	Anti-trypanosomal activity, high toxicity (mice LD_50_: 0.2 mg kg^−1^)	25
Complex nucleoside antibiotics	Miharamycin/amipurimycin	Inhibitors of *P. oryzae* (rice blast disease), MIC amipurimycin: 5 μg mL^−1^	26 and 27
	A201A	Active against Gram-positive bacteria, MIC: 1–8 μg mL^−1^	28

a LD = lethal dose, values for injections are given; CD = curative/protective dose; GMP = guanosine monophosphate; SAH = *S*-adenosylhomocysteine; MIC = minimal inhibitor concentration.

We have organised these natural products into different sub-classes and consider each individually within a framework of forward chemical synthesis and biosynthetic perspectives. The structural classifications covered are highlighted in Fig. 1.

## 2. Purine *C*-nucleosides

### 2.1. Enzymes involved in the biosynthesis of purine *C*-nucleosides

**Fig. 2:**
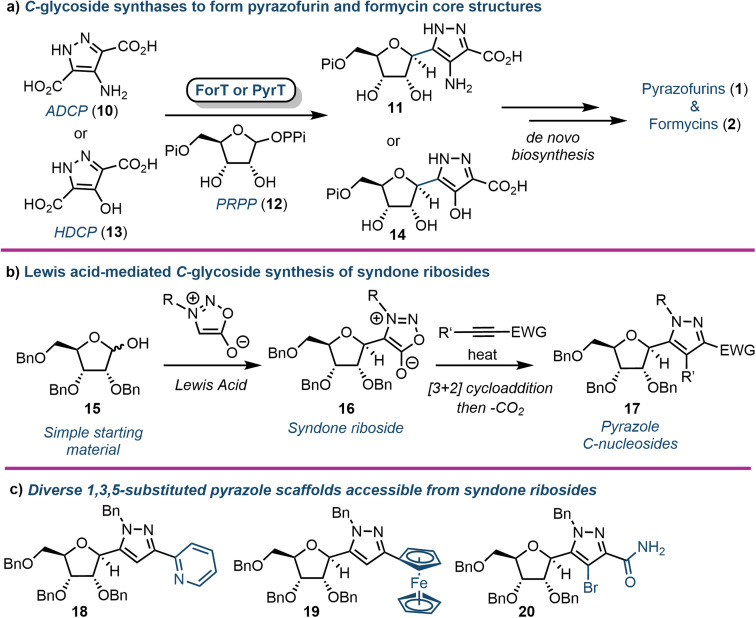
(a) Biosynthesis of pyrazofurin and formycin cores using the *C*-glycoside synthases ForT or PyrT. (b) Chemical synthesis of pyrazole *C*-nucleosides *via* syndone ribosides (16); (c) examples of 5-position diversity accessible using this approach; ADCP (10) = 4-amino-1*H*-pyrazole-3,5-dicarboxylate, HDCP (13) = 4-hydroxy-1*H*-pyrazole-3,5-dicarboxylate, PRPP (12) = phosphoribosyl pyrophosphate, Pi = phosphate, PPi = pyrophosphate.

*C*-Nucleosides are produced in nature and are characterised by an unusual carbon–carbon bond between the sugar backbone and the nucleobase (relative to the native β-*N*-glycosidic linkage). As a result, these nucleosides cannot be degraded by standard salvage pathway enzymes (*e.g.*, nucleoside phosphorylases or hydrolases).^29^ This catabolic stability makes them an interesting target for drug development. The biosynthetic pathways for the purine *C*-nucleosides pyrazofurin (1) and formycin (2) have only recently been elucidated. Liu and colleagues first identified the gene cluster of 2 in *S. kaniharaensis* using intensive cosmid library screening in 2019.^30^ From this seminal work the same group, and alongside the Chen group, independently showed that ForT from *S. candidus* and PyrT (also named PyfQ) from *S. kaniharaensis* are *C*-glycoside synthases (Fig. 2a).^31,32^ ForT and PyrT were formally annotated as β-ribofuranosylaminobenzene 5′-phosphate synthase-like enzymes (β-RFA-P, EC 2.4.2.54), which are known from the biosynthesis of methanopterin, a cofactor component in methanogens (anaerobic archaea).^33^ Notably, ForT is involved in the formycin pathway and PyrT in pyrazofurin biosynthesis.

Heterologously expressed ForT or PyrT incubated with phosphoribosyl pyrophosphate (PRPP) and 4-amino-1*H*-pyrazole-3,5-dicarboxylate (ADCP) (10) or 4-hydroxy-1*H*-pyrazole-3,5-dicarboxylate (HDCP) (13) respectively, led to the formation of the corresponding carboxypyrazole ribo-*C*-nucleotides 11 or 14 (Fig. 2a). Furthermore, the crystal structure of ForT in complex with PRPP (12) has recently been resolved to 2.5 Å (PDB ID: 6YQQ), providing the location of active site residues critical for the recognition of 12 and catalysis, paving the way for further biocatalytic development and application of such *C*-glycoside synthases.^34^

### 2.2. Syndone ribosides as a synthetic entry point to diverse purine *C*-nucleosides

In 2020 Van Calenbergh and colleagues disclosed a platform to access pyrazole *C*-nucleosides,^35^ including a synthesis of pyrazofurin and formycin B. The work focused on establishing access to syndone ribosides (16) (Fig. 2b), which were subjected to a [3 + 2] dipolar cycloaddition with an alkyne partner, followed by extrusion of CO_2_*via* a retro-Diels–Alder reaction. This afforded *N*-protected-5-substituted pyrazoles (17) with multiple orthogonal diversification points. The approach began by attempting nucleophilic addition to a C1 ribonolactone, followed by anomeric dehydrosilylation to deliver the β-syndone riboside preferentially. However, this process was not efficient, and the authors instead pursued a direct Lewis acid-mediated *C*-glycosylation. This proved effective, albeit delivering α/β mixtures of syndone ribosides that required separation before cycloaddition could take place. Several different alkyne coupling partners were then explored to deliver a library of *C*-linked pyrazole analogues, demonstrating capability to pursue future structure–activity-relationship studies around this motif (Fig. 2c). The utility of the approach was further demonstrated by completing formal syntheses of formycin B and pyrazofurin.

## 3. Purine nucleosides incorporating ribose ring modifications

The angustmycin and aristeromycin families of purine nucleosides are potent antibiotics, inhibiting the synthesis of guanosine monophosphate (GMP) in Gram-positive bacteria and *S*-adenosyl homocysteine (SAH) hydrolases in rice blast diseases.^20–23^ They have been prepared both enzymatically and chemically; recent discoveries towards both families are considered sequentially here.

### 3.1. Angustmycins A & C

**Fig. 3:**
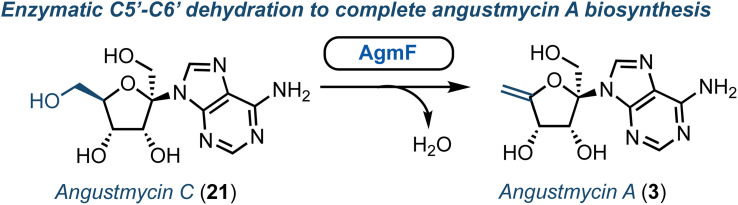
Identification of AgmF to access angustmycin A (3) from angustmycin C (21).

Structurally, the angustmycins are analogues of adenosine (8), but contain an unusual six carbon ketose (β-d-psicofuranose), incorporating an additional α-hydroxymethyl group at the 1′-position. Angustmycin A (3) differs from angustmycin C (21) by an *exo*-5′,6′-alkene in place of the 5′-hydroxymethyl substituent (Fig. 3). Independently, the groups of Kuzuyama and Price & Chen reported the identification of the angustmycin biosynthetic gene cluster in 2021.^36,37^ They identified and showed *in vitro* that AgmF (annotated as an SAH hydrolase, EC 3.13.2.1) was an unusual dehydratase that transformed 21 into 3 (Fig. 3). Additionally, it was shown by Price and Chen that AgmF could maintain its activity without the addition of NAD^+^, indicating an intriguing self-sufficiency for co-factor recycling. By heat-treating the enzyme, NAD^+^ was released suggesting that the cofactor was bound tightly within the active-site pocket. Further point mutations of the protein confirmed six amino acid residues to be critical for binding NAD^+^ close to the catalytically active Lys-185. Finally, the whole biosynthesis pathway was both reconstructed in *E. coli in vivo* and with purified enzymes *in vitro* to deliver a one-pot synthesis of 3. Whilst 3 and 21 have both been prepared chemically (*e.g.*, starting from d-fructose),^38^ this recent advancement from a biocatalytic perspective may surpass such capability, as it offers a direct entry point to produce these scaffolds for wider chemical or enzymatic diversification.

### 3.2. Aristeromycin

**Fig. 4:**
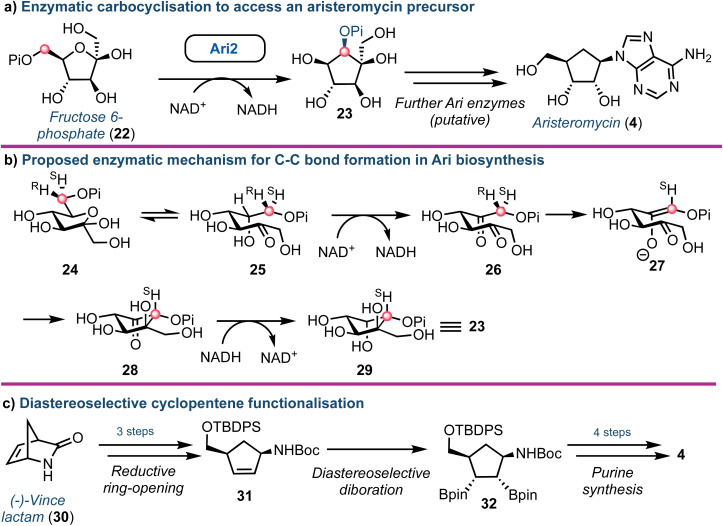
(a) Identification of Ari2 catalysing the rearrangement of fructose 6-phosphate (22) to a cyclopentane core (23). Red dot tracks C6 through the skeletal rearrangement. (b) Proposed mechanism for the formation of the cyclopentane core 23.^41^ (c) Overview of a recent chemical synthesis of aristeromycin (4), starting from a non-carbohydrate chiral pool material.

Aristeromycin (4) differs from canonical systems through the purine base being bound to a cyclopentane ring (in place of d-ribofuranose). Such analogues are known substrates and inhibitors of SAH hydrolases.^39^ However, their biosynthesis and especially the cyclisation step from cyclic ketose to a polyhydroxylated cyclopentane, is intriguing. The Eguchi group recently demonstrated that the enzyme Ari2 utilises fructose 6-phosphate as a donor to produce a phosphorylated six carbon product (Fig. 4a).^40^ Following heterologous expression of Ari2 in *E. coli* and purification *via* affinity chromatography, the structure of the five-membered cyclitol phosphate product was confirmed using NMR; the relative stereochemistry of the cyclopentane system was supported using n*O*e analysis. Furthermore, ^31^P NMR confirmed NAD^+^ as the cofactor for Ari2 and that this could not be replaced by NADP^+^. Ari2 from *S. citricolor* is a myo-inositol-1-phosphate (MIP) synthase ortholog (EC 5.5.1.4) and recently the same group elucidated the stereochemistry of this critical reaction. The authors fed 6*R*- or 6*S*-(6-^2^H) glucose (24) to an *S. citricolor* fermentation culture and established a diastereoselective proton abstraction from the C6 position within fructose 6-phosphate (as part of the mechanism to form the new C–C bond), confirming that Ari2 operates in a MIP synthase fashion (proposed mechanism highlighted in Fig. 4b).^41^

From a chemical synthesis perspective, successful procedures have been developed from carbohydrate starting materials such as d-ribose.^42^ More recently an alternative starting from (−)-2-azabicyclo[2.2.1]hept-5-en-3-one 30 (Vince lactam, Fig. 4c) was reported by Morken.^43^ When the lactam nitrogen was protected with an electron withdrawing carbamate protecting group (such as *tert*-butyloxycarbamoyl, Boc), subsequent reduction of the cyclic amide using NaBH_4_ was enabled, affording a *cis*-disubstituted cyclopentene 31. Using a Pt(dba)_3_ catalyst, selective diboration of the internal alkene using B_2_(pin)_2_ was accomplished *trans* to the *cis* functional groups affording diborinate 32. The borinate groups were then converted into their respective hydroxyl groups using H_2_O_2_. Overall, this diastereoselective alkene functionalisation bypassed the need for hazardous oxidants and the material was converted into 4, building the adenine base from the pseudo-anomeric position.

## 4. Halogenated purine nucleosides

**Fig. 5:**
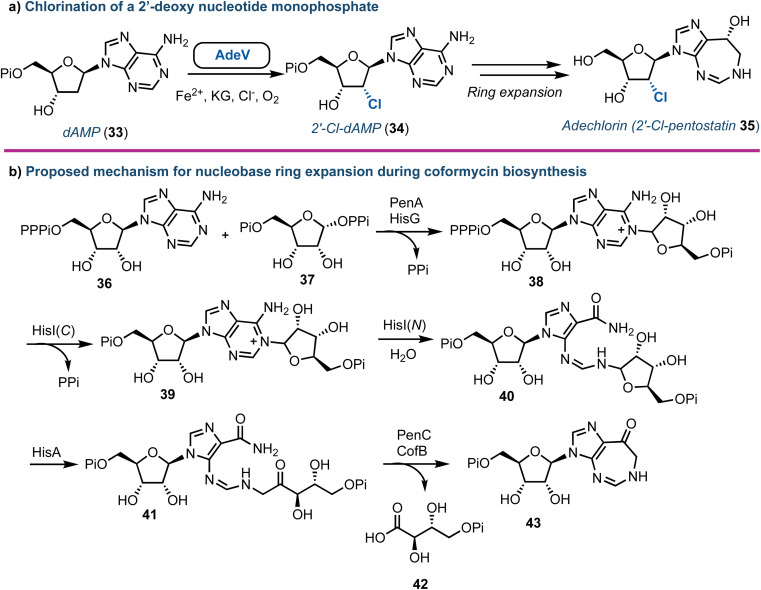
(a) Discovery of AdeV to complete stereospecific 2′-chlorination of 2′-deoxyadenosine monophosphate. (b) Proposed mechanism for the enzymatic ring expansion during the biosynthesis of coformycin.^52^ dAMP (33) = 2′-deoxyadenosine monophosphate; Pi = phosphate, PPi = pyrophosphate, PPPi = triphosphate.

Halogenated nucleoside analogues are commonly encountered as antiviral and anticancer drugs, exemplified by sofosbuvir and cladribine. In contrast to these pharmaceuticals, naturally occurring halogenated nucleosides are rare.^44^ Excitingly, in 2020 Deng and Zhang revealed the first Fe^2+^-α-ketoglutarate dependent halogenase to act upon a 2′-deoxy nucleotide framework.^45^ Through re-sequencing the *Actinomadura* genome, AdeV was found and the corresponding protein was shown to catalyse the conversion of 2′-deoxyadenosine-5′-monophosphate (dAMP, 33) into 2′-deoxy-2′-Cl-dAMP (34) *in vitro* (Fig. 5a). This finding was key, as although the biosynthesis of adechlorin (35) had been deciphered,^46,47^ the responsible chlorinase was still unknown. Intriguingly, AdeV showed only 11% amino acid similarity to Wel05, a nonheme iron enzyme, known to chlorinate alkaloids, including 12-*epi*-hapalindole C.^48,49^ Recent crystal structure and point mutation studies have provided insight into the molecular mechanisms for these enzymes [PDB IDs: 7W5T (1.76 Å), 7V57 (2.35 Å)] , paving the way for future enzymatic synthesis of halogenated nucleosides.^50,51^ Furthermore, the group of Liu have elucidated the ring-expanding steps *in vitro* occurring during coformycin biosynthesis to access the unusual 1,3-diazepine nucleobase (Fig. 5b).^52^

**Fig. 6:**
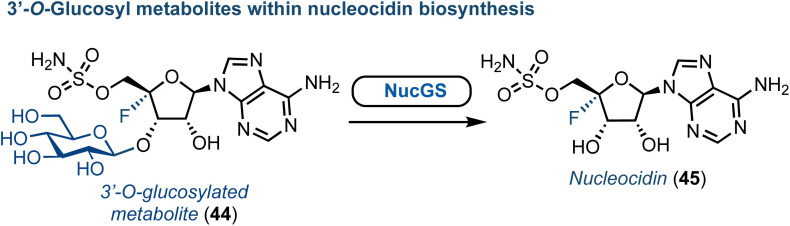
Discovery of a glucosylated metabolite (44) within nucleocidin biosynthesis.

Advances towards understanding the biosynthesis of nucleocidin (45) (a 4′-fluorinated nucleoside, Fig. 6) is another notable recent success concerning the origin of halogenated purine nucleoside antibiotics. First discovered in *S. calvus* in 1951, this compound has regained new interest due to its remarkable biological and structural profile.^53^ The nucleocidin biosynthetic cluster has been identified and the enzymes catalysing the early steps of biosynthesis characterised.^54^ More recently, gene disruption experiments by the Zechel and O'Hagan groups have independently provided clues towards the enzymatic requirements for 4′-fluorination and 5′-sulfamate ester formation.^55–57^ Furthermore, an unusual 3′-*O*-β-glucosylated metabolite was identified by O'Hagan (Fig. 6).^58^ Therefrom, a UDP-Glc dependant glycosyl transferase (NucGT) and a glucosidase (NucGS) were uncovered and shown to be able to functionalise the 3′-position of adenosine derivatives. Intriguingly, the glucosidase could generate 45, suggesting the 3′-*O*-glucosylated substrate may be the product of the fluorination step. However, the biochemical mechanism for 4′-C–F bond formation and the specific enzyme performing this halogenation step remains unclear. The chemical synthesis of 45 was established in the 1970s,^59^ and has been reviewed recently elsewhere.^53^

## 5. Complex purine nucleoside antibiotics

### 5.1. Peptidyl purine nucleosides

**Fig. 7:**
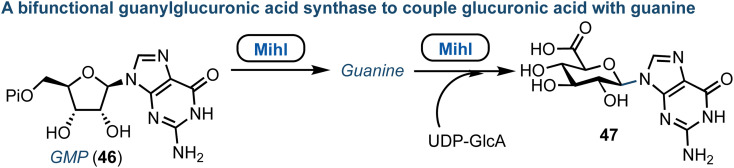
Discovery of MihI to complete ribose to glucuronic acid transglycosylation with retained β-anomeric stereochemistry; Pi = phosphate.

The core structure of purine nucleoside antibiotics can be significantly modified by nonribosomal peptide synthases (NRPS) and polyketide synthases (PKS). The miharamycins and amipurimycin are exemplar systems, containing a C9 pyranosyl core and a 2-aminopurine. They are produced by *S. miharaensis* and *S. novoguineensis* respectively and recent reports from the groups of Liu and Tang independently identified the responsible biosynthetic gene cluster.^60,61^ Their studies indicated that a polyketide synthase catalyses the early steps of biosynthesis for both miharamycin and amipurimycin, towards the high-carbon sugar core, and that within this MihI acts as bifunctional guanylglucuronic acid synthase (Fig. 7).^62^ MihI is responsible first for the anomeric hydrolysis of GMP (46), followed by use of the released guanine for the formation of a new C–N bond *via* reaction with the sugar nucleotide donor, UDP-GlcA. This reaction is particularly interesting as it incorporates a pyranose in place of ribose. Furthermore, C–N bond formation in nucleosides is usually catalyzed by nucleoside phosphorylases or phosphoribosyl transferases.

**Fig. 8:**
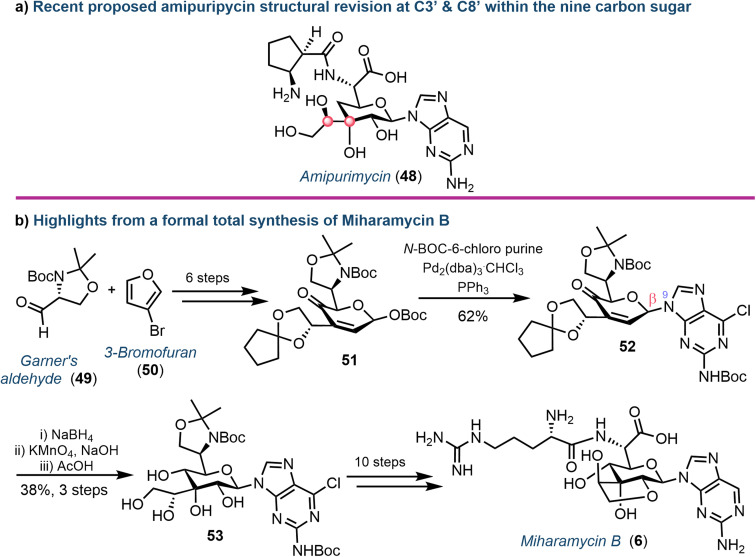
(a) Proposed structural revision to amipurimycin (48) concerning stereochemical assignments at C3′ and C8′ of the sugar (highlighted with red dots). (b) Overview of key steps from the total synthesis of miharamycin B (6).

Yu and colleagues recently described the first total synthesis of amipurimycin (48), completing a 27-step process that adopted a convergent strategy, coupling the C9 pyranose core to the respective amino acid and 2-amino purine components.^63^ Importantly, a comparison of the NMR data from the isolated natural product to those obtained from previous syntheses highlighted inconsistencies,^64^ and led to the authors proposing a revised configurational assignment at C3′ and C8′ of this natural product (Fig. 8a), supported using X-ray crystallography.^63,65^

More recently, the chemical synthesis of miharamycin B (6) and analogues was reported by Wang and Liu.^66^ Starting from enantiopure (*R*)-Garner's aldehyde (49) and 3-bromofuran (50), a 20-step asymmetric *de novo* approach was designed (Fig. 8b). Notable from this was coupling of a pyranose-derived intermediate (51) with *N*-Boc-C6-chloropurine using palladium. The choice of the C6-chloropurine nucleophile proved critical for obtaining the required *N*^9^-regioselectivity; the use of C6-bromo or iodo purines resulted in oxidative addition of Pd to the carbon halogen bond, competing with the activation of the allylic *O*-Boc electrophile. The stereocentres within the remaining sugar framework were installed through reduction of the C4′-ketone (using NaBH_4_) followed by stereocontrolled dihydroxylation of the C2′–C3′ alkene using KMnO_4_ to afford 53. Finally, removal of the C8′–C9′-acetonide protecting group furnished the desired core structure and enabled completion of the total synthesis for 6: a series of protecting group manipulations, cyclisation to form the fused ring system, coupling of arginine and dehalogenation of the nucleobase.

### 5.2. Aminonucleoside natural products

Another important class of complex purine nucleoside antibiotics is the A201 family. Consisting of a 3′-deoxy-3′-amino adenosine core, a derivative from this family, A201A (7), was first isolated from *S. capreolus*,^67^ and further steps to elucidate the biosynthetic pathway in *M. thermotolerans* (largely concerning the sugar components) have been completed by Ju.^68^

**Fig. 9:**
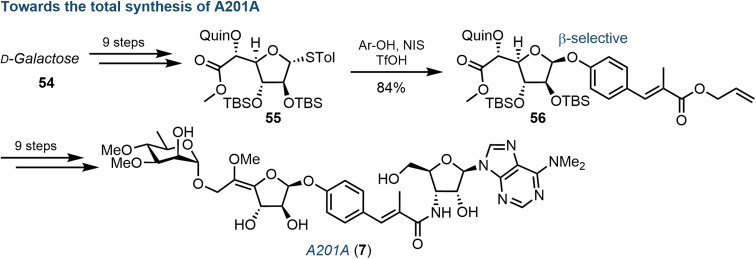
Overview of chemical synthesis of A201A (7), highlighting a key diastereoselective β-glycosylation of a galactofuranosyl donor (55); Quin = quinoline ester.

Wang *et al.* recently reported a total chemical synthesis of the A201 family, using a convergent strategy reliant on the coupling of three carbohydrate-based building blocks: a galactofuranose, a 3′-amino adenosine and a d-rhamnose.^69^ Underpinning to this synthesis was stereoselective installation of the 1,2-*cis* glycosidic linkage to the central galactofuranose subunit (Fig. 9). The use of a quinoline ester at the C5-position proved invaluable, allowing for an elegant β-directed, Lewis-acid mediated glycosidation of a phenolic acceptor, proposing coordination of the phenol moiety to the quinoline nitrogen and affording intermediate 56. With the desired linkage in place, the remaining synthesis of 7 was completed over 9 steps, including: *Z*-selective formation of the exocyclic methoxy enol ether, coupling of the 3′-amino adenosine moiety and attachment of the remaining 3,4-di-*O*-methylated rhamnose.

## 6. Conclusion and outlook

Natural products offer a rich source of structural diversity to discover new nucleoside analogues. Small modifications to such structures can build structure–activity relationships and probe new druggable space. Classically, following their isolation and structural characterisation, total chemical syntheses of such molecules would be completed to obtain relevant quantities of material for further or wider biological investigation. However, as illustrated herein, the length and complexity of some chemical syntheses (*e.g.*, 27 steps in the case of 48) demands a substantial amount of resource. It is here that a synergy of biology (isolation, understanding and recombinant production of biosynthetic enzymes) and chemistry (synthesis and structural analysis) may prove transformative in facilitating the future of purine nucleoside antibiotic discovery. Indeed, such concepts can be drawn from examples illustrated herein. An ability to access proteins that can affect intriguing and chemically difficult transformations – stereospecific 2′-chlorination (AdeV), furanose to pyranose transglycosylation (MihI), carbocyclisation (Ari2) – could rapidly access key core materials for further derivatisation. Furthermore, the identification of new metabolic enzymes from within nucleocidin biosynthesis provides opportunities to explore such derivatives and related systems, such as the 7-deazapurine tubercidin.^70,71^

As our ability to make mimetic protein structures progresses, an integration of synthetic biology within classical purine nucleoside chemical synthesis will be transformative.^72^ Entering a post-antibiotic era predicates a need to develop new therapeutics. In this context, purine-derived nucleoside antibiotics are a promising and largely untapped source of potential. There is a fascinating diversity to this compound class and several examples have already demonstrated promising antibacterial activities.^2,73^ Realising their potential will rely on the continued harmony of biocatalysis with chemical synthesis,^74^ a multidisciplinary approach already delivering across the wider field of natural product synthesis.^75^ In recent years, the biocatalytic synthesis of nucleosides and derivatives has burgeoned, for example by exploiting the promiscuity and thermostability of nucleoside phosphorylases,^76–78^ and such strategies could be envisaged to further expand and ease the synthesis of purine nucleoside antibiotics.

## 7. Author contributions

Conceptualization: J. M. and P. N.; writing – original draft: J. M., C. B., P. S.-B. and G. M. writing – review & editing: J. M., C. B., S. W., P. S.-B., P. N., A. K. and G. M.; visualization: J. M. and G. M.; supervision: P. N., A. K. and G. M.; project administration: P. N., A. K, and G. M.

## 8. Conflicts of interest

AK is CEO of BioNukleo GmbH. SW is a scientist at BioNukleo GmbH. PN is a member of the BioNukleo GmbH advisory board. These affiliations constitute no conflict of interest with the topics presented and discussed in this report. The remaining authors declare no conflict of interest.
